# Refractive Outcomes of Non-Toric and Toric Intraocular Lenses in Mild, Moderate and Advanced Keratoconus: A Systematic Review and Meta-Analysis

**DOI:** 10.3390/jcm11092456

**Published:** 2022-04-27

**Authors:** Tal Yahalomi, Asaf Achiron, Idan Hecht, Roee Arnon, Eliya Levinger, Joseph Pikkel, Raimo Tuuminen

**Affiliations:** 1 Department of Ophthalmology, Samson Assuta Ashdod Hospital, Faculty of Health Sciences, Ben-Gurion University of the Negev, Beer-Sheva 7747629, Israel; yatal25@gmail.com (T.Y.); roee.arnon@gmail.com (R.A.); yossefp@assuta.co.il (J.P.); 2 Department of Ophthalmology, Tel-Aviv Sourasky Medical Center, Tel-Aviv 6423906, Israel; achironasaf@gmail.com (A.A.); eliya.levinger@gmail.com (E.L.); 3 Sackler Faculty of Medicine, Tel-Aviv University, Tel-Aviv 6997801, Israel; idanhe@gmail.com; 4 Department of Ophthalmology, Shamir Medical, Zerifin 7033001, Israel; 5 Department of Ophthalmology, Kymenlaakso Central Hospital, 48210 Kotka, Finland; 6 Helsinki Retina Research Group, Faculty of Medicine, University of Helsinki, 00014 Helsinki, Finland

**Keywords:** biometry, intraocular lens power calculation formula, keratoconus, refractive error, spherical equivalent

## Abstract

Background: To perform a systematic review and meta-analysis of the refractive outcomes of non-toric and toric intraocular lenses (IOLs) in keratoconus (KC) using different IOL power calculation formulas. Methods: A systematic search was conducted to identify studies that report on refractive outcomes of different IOL power calculation formulas in KC patients undergoing cataract surgery. Inclusion criteria were primary posterior chamber non-toric and toric monofocal intraocular lens implantation, data on the degree of KC, explicit mention of the formula used for each stage of KC, and the number of eyes in each category. We calculated and compared the absolute and mean prediction errors, percentage of eyes within 0.5 D and 1 D from target, and the weighted absolute prediction errors of IOL formulas, all were given for KC degrees I–III. Results: The bibliographic search yielded 582 studies published between 1996 and 2020, 14 of which (in total 456 eyes) met the criteria: three studies on non-toric IOL (98 eyes), eight studies on toric IOLs (98 eyes) and three studies of unknown separation between non-toric and toric IOLs (260 eyes). The lowest absolute prediction error (APE) for mild, moderate, and advanced KC was seen with Kane’s IOL power formula with keratoconus adjustment. The APE for the top five IOL power formulas ranged 0.49–0.73 diopters (D) for mild (83–94%) of eyes within 1 D from the target), 1.08–1.21 D for moderate (51–57% within 1 D), and 1.44–2.86 D for advanced KC (12–48% within 1 D). Conclusions: Cataract surgery in eyes with mild-to-moderate KC generally achieves satisfactory postoperative refractive results. In patients with advanced KC, a minority of the eyes achieved spherical equivalent refraction within 1 D from the target. The Kane’s formula with keratoconus adjustment showed the best results in all KC stages.

## 1. Background

Keratoconus (KC) is the most common corneal ectasia. Depending on the definition and geographic location the estimated prevalence of KC ranges from 0.17 to 40 in 1000 inhabitants [1,2,3]. Planning cataract surgery for patients with KC is challenging, as the refractive outcome could be difficult to predict. Currently, there is no consensus on the best intraocular lens (IOL) formula in terms of accuracy in KC patients [4,5,6,7].

Proper refractive outcomes carry major implications for vision-related quality of life in KC patients. Modern surgical techniques and equipment have decreased the intraoperative complications associated with the surgery and improved the treatment results [8]. Nevertheless, achieving the best outcomes in postoperative visual function requires diligent planning and should take into account individual patient characteristics.

The refractive results of most non-keratoconus eyes after cataract extraction and IOL implantation are usually satisfactory with the estimation of 80% of eyes within 0.5 D of refractive target [9], while the results of keratoconus eyes are often worse for several reasons. First, the IOL power calculations assume the ratio of the anterior to posterior corneal power, which is not preserved in keratoconus eyes [10,11]. Second, the IOL power calculations use the corneal power in the effective lens position (ELP) estimation, which means a false evaluation [12,13]. Third, in keratoconus eyes the corneal curvature is often variable in a specific meridian making the corneal keratometric power meridian measurements non-orthogonal [14].

The IOL formulas are optimized for the normal eye that does not resemble the KC model. There are guidelines that clarify which IOL calculations are best suited for KC patients and under what terms; however, to the best of our knowledge, there is no current systematic review or meta-analysis on this subject. Ghiasian et al. published in 2019 a study on cataract surgery in KC patients, emphasizing challenges encountered during IOL power calculation and their solutions, and concluded that SRK II formula might provide the most accurate IOL power in mild KC [15]. In contrast, the literature on moderate and severe KC is limited. In our meta-analysis, we incorporated Kane keratoconus formula and addressed all KC stages comparing toric and non-toric IOLs [16]. Kane et al. compared various IOL calculation formulae in their retrospective consecutive case series on 147 eyes and discovered that the Kane keratoconus formula had significantly decreased the median absolute error (MedAE) compared to all formulas. 

The scope of the study was to perform a systematic review and meta-analysis of the refractive outcomes of non-toric and toric intraocular lenses (IOLs) in keratoconus (KC) using different IOL power calculation formulas and in different disease stages.

## 2. Materials and Methods

### 2.1. Literature Search Criteria

A systematic search was conducted using Cochrane Library and MEDLINE, PubMed, ClinicalTrials.gov, metaRegister of Controlled Trials (www.controlled-trials.com, accessed on 19 May 2020), WHO International Clinical Trials Registry Platform (http://www.who.int/ictrp/search/en, accessed on 19 May 2020), and Google Scholar with the following keywords: keratoconus, intraocular lens (IOL) calculation, biometry, cataract. Additional records were identified by hand-searching the bibliographies of relevant studies. We have included prospective and retrospective studies and case reports meeting the inclusion criteria.

Here we aimed to identify studies that relate to the effects of different IOL power calculation methods in stable keratoconus patients undergoing cataract surgery with monofocal non-toric or toric IOL implantation. The patients chosen were without any ocular morbidity, glaucoma or retinopathy, no previous trauma or ocular inflammation, without central corneal scars, and no prior cross-linking therapy. Any patient must be present with a stable and non-progressing keratoconus proven by visual and topographic characteristics. We included studies meeting the following criteria: (i) the study population included a keratoconus group, (ii) the study examined any objective outcome measure relating to IOL calculation (e.g., BCVA, refraction outcome, visual activities of daily living), (iii) the study was published in English, (iv) full-length original articles were included (not an Abstract or Letter to the Editor), and (v) the study was published between 1980 and 2020 (Table 1, Supplementary Materials Figure S1). 

The review process was conducted under the guidance of the PRISMA (Preferred Reporting Items for Systematic Reviews and Meta-Analyses) criteria to support reporting [17]. Two investigators (T.Y., A.A.) independently searched for relevant publications. The selected publications were then approved by a senior investigator (J.P.). Individual studies were graded using the Scottish Intercollegiate Guideline Network (SIGN) assessment system for individual studies as implemented for Preferred Practice Patterns by the American Academy of Ophthalmology (AAO) [18] (Table 1, Supplementary Materials Figure S1). 

### 2.2. Patients and Definitions

Patients with keratoconus undergoing cataract surgery with posterior chamber intraocular lens (PCIOL) implantation were included. 

Specifically, the inclusion criteria were as follows: (i) KC patients undergoing cataract surgery, (ii) the degree of KC according to keratometry and Krumeich criteria [19] for classification of keratoconus, (iii) explicit mention of IOL power calculation formula for each degree of KC, and (iv) the number of eyes in each category were mentioned. Exclusion criteria were surgery for phakic IOL or combined with refractive, pars plana vitrectomy surgery or intra-corneal ring segment implantation. 

The eyes were classified following the Krumeich criteria [19] belonging to stage I–IV (Supplementary Materials Table S1). The Krumeich criteria was chosen as it is the most common classification method for KC eyes. No eyes belonging to stage IV (unmeasurable refraction or with a central scar) were included in this study. 

**Table 1:** Summary of studies.

Study	Year	Country	Number of Eyes	Follow-Up	Design	Strength of Evidence ^a^
Thebpatiphat et al. [6]	1996	USA	12	3 months	A retrospective case series	III
Kane et al. [16]	2007	Australia	146	NA	A retrospective case series	III−
Celikkol et al. [20]	2013	USA	2	6 weeks	A case report	III
Watson et al. [21]	2009	UK	84	1–116 months (mean 33 months)	A retrospective case series	III
Navas et al. [22]	2011	Mexico	2	5 years	A case report	III
Visser et al. [23]	2011	Netherlands	3	6 months	A case report	III
Jaimes et al. [24]	2012	Mexico	19	3–31 months (mean 7.89 months)	A retrospective case series	III
Nanavaty et al. [25]	2013	UK	12	mean 9 months	A retrospective case series	III
Parikakis et al. [26]	2014	Greece	5	18–28 months	A case report	III
Alió et al. [27]	2015	Spain	15	3–15 months (9.1 months)	A retrospective case series	III
Hashemi et al. [28]	2016	Iran	23	3 months	A retrospective case series	III
Kamiya et al. [29]	2019	Japan	19	3 months	A prospective study	II
Savini et al. [30]	2020	Italy	41	One month	A retrospective case series	III
Wang et al. [31]	2020	USA	73	NA	A retrospective case series	III

^a^ Strength of evidence was graded using the Scottish Intercollegiate Guideline Network (SIGN) assessment system for individual studies as implemented for Preferred Practice Patterns by the American Academy of Ophthalmology [17]. For example: I = meta-analysis, systemic reviews of RCT or RCT; II = systemic reviews, case–control or cohort studies; III = case reports or case series. + and − signs designate risk of confounding or bias. NA; not available.

### 2.3. Statistical Analysis

Meta-analyses were performed using the Cochrane Collaboration Review Manager Software version 5.3.5 (Cochrane Collaboration, Oxford, UK). 

We calculated and compared between the absolute and mean prediction errors (PE; the absolute difference between the predicted and the observed refraction, expressed in spherical equivalent in diopters), percentage of eyes within 0.5 D and 1 D from target and the weighted absolute prediction errors of IOL formulas, all were given for KC degrees I–III.

For continuous variables, a summary table was provided, with arithmetic means and standard deviations. Paired-sample *t*-tests were applied to assess differences. *p*-value ≤ 0.05 was considered statistically significant. The data were analyzed using the SPSS version 27 (SPSS Inc., Chicago, IL, USA).

## 3. Results

The bibliographic search yielded 582 previous studies that were filtered and reviewed. A large number of studies did not meet the inclusion criteria. Moreover, we reviewed each article’s reference bibliography to cover further studies that had not been identified earlier. As a result of this two-step process, data that met our criteria were extracted from 14 publications and analyzed for the study’s main questions (Supplementary Materials Table S2). 

Included were 14 studies published between 1996 and 2020 on 456 eyes, 3 studies concerning non-toric IOLs (98 eyes), 8 studies on toric IOLs (98 eyes), and 3 studies with unknown separation between non-toric and toric IOLs (260 eyes) (Table 1). No study directly compared the non-toric and toric IOL refractive results. In total, 269 eyes (59%) were KC stage I based on Krumeich criteria, 138 eyes (30%) were stage II, and 49 eyes (11%) were stage III.

Seven articles directly compared different IOL power formulas and the rest reported on visual outcomes using only one formula. Three articles originated from the USA, six from Europe, two from Mexico, one from Iran, one from Japan, and one from Australia. The most common formula used was SRK/T in 11 studies; followed by Hoffer Q used in 6 studies; Holladay I in 5 studies; SRK II in 4 studies; and SRK, Holladay II, Haigis, Olsen, Barrett, and Kane in 3 or fewer studies. 

Absolute and mean prediction errors, percentage of eyes within 0.5 D and 1 D from target are presented for stage I [6,16,20,21,22,23,24,25,26,27,28,29,30,31] (Table 2), stage II [16,20,21,24,25,27,28,29,30,31] (Table 3), and stage III [16,21,28,30,31] (Table 4). 

**Figure 1:**
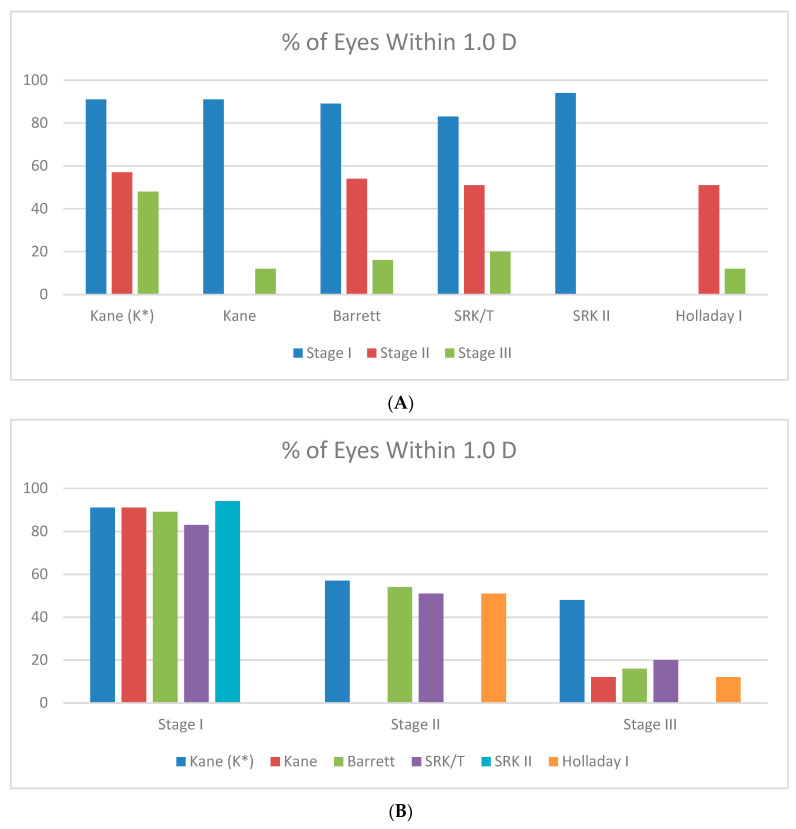
(**A**) Percentage of eyes within 1.0 D in accordance to IOL formula; (**B**) Percentage of eyes within 1.0 D in accordance to the Keratoconus stage.

Finally, weighted absolute prediction errors (APEs) of IOL formulas and percentage of eyes within 0.5 D and 1 D from target were given for KC degrees I–III (Table 5). Of the studies reporting on patients with mild KC (stage I), APE for the top five formulas ranged between 0.49 and 0.73 D, with 83–94% of eyes being within 1 D from the target. The respective values were 1.08–1.21 D and 51–57% for moderate KC (stage II), and 1.44–2.86 D and 12–48% for advanced KC (stage III). Figure 1A,B represents percentage of eyes within 1.0 D in accordance to IOL formula or keratoconus stage, respectively.

## 4. Discussion

This study examined different IOL calculation formulas in eyes with KC of different stages. The results show that in mild-to-moderate KC undergoing cataract surgery a satisfactory postoperative refractive result is generally seen. On the other hand, patients with advanced KC showed less predictable postoperative refractive results regardless of the formula used [6,30]. The challenge in KC patients undergoing cataract surgery is to maximize visual potential; however, achieving desirable refraction could have dramatic impacts on quality of life.

The first IOL power calculation formulas employed axial length, corneal refractive power and the predicted postoperative anterior chamber depth. Newer-generation formulas include the vergence and effective lens position and further modifications have been made to vergence-based IOL formulas [32]. The predictability of refractive outcomes after cataract surgery in general has improved significantly [9,33]. Newer-generation formulas have been validated in various studies and reported with up to 88% and 97.8% of eyes achieving a postoperative spherical equivalent (SE) refraction within ±0.5 and ±1.0 D, respectively [33,34,35,36].

As with the management of any patient with KC, timing of corneal cross-linking (CXL) should be considered. Some authors suggest that CXL should be performed prior to cataract surgery [37], while some others suggest avoiding CXL due to the tendency of ectasia to be stable above the age of 50. A two-stage procedure of clear lensectomy with toric IOL implantation after CXL has been reported with satisfactory outcomes in selected cases of keratoconus [38]. To maximize the accuracy of the IOL power, Leccisotti et al. suggested the use of an intraoperative autorefractometer in severe cases of KC [7]. Furthermore, the position of the corneal incision should also be taken into account as the main corneal incisions should not be placed according to the astigmatism axis, but according to the peripheral corneal thickness [4,39]. 

The study of Savini et al. concluded that, in general, the best outcomes in KC eyes were achieved with the SRK/T formula which was even better than the third and fourth generation formulas [30]. The SRK/T formula achieved the lowest median absolute error and the highest rate of eyes with prediction error within ±0.50 D [30]. In the present study, we hypothesized that the appropriate IOL formula might depend on the stage of KC. Interestingly, Kane’s keratoconus adjustment formula outperformed in all KC classification stages, especially in the advanced stage III KC eyes. In mild KC, all top five IOL power calculation formulas yielded high rates of eyes (around 90%) within 1D of target, while in moderate and advanced KC the respective levels after primary IOL implantation were up to 57% and 48% of eyes. In accordance with the literature and our clinical experience, we believe that mild residual myopia is the most appropriate refraction aim for KC patients undergoing cataract surgery. The study of Savini et al. found that, in the whole sample (41 eyes), the mean prediction error (PE) was positive (hyperopic surprise) with all formulas; the lowest PE and MedAE were obtained with the SRK/T formula [30]. Aiello et al. stated that a low myopic post-operative refractive outcome is advantageous to a hyperopic refractive outcome not only for unassisted near vision, but also for scleral CL use [4]. The pre-corneal tear fluid reservoir often functions as an extremely high power minus lens, occasionally reaching –15.00 D. As a result, the adoption of scleral CLs may be more problematic in cases with postoperative hyperopic refractive outcome. This is due to the fact that positive powered scleral CLs are less acceptable, necessitating bigger central thicknesses with smaller front optic zone diameters, resulting in a CL that is generally more mobile in situ and has higher degrees of optical aberration [4]. Kane et al. suggested no changes to the target aim in stage I, a more myopic objective (between −0.75 and −1.5 D) in stage II, and even more myopic target (between −2.0 and −3.0 D) in stage III keratoconus [16]. These modifications are less drastic than those proposed by Watson et al. [21]. Recommendations of the abovementioned studies for refractive aims are detailed in Supplementary Materials Table S2.

The Kane formula is a relatively new IOL power formula that was developed using parameters such as the patient’s axial length, keratometry, anterior chamber depth, lens thickness, central corneal thickness, and gender [40]. This formula was based on large datasets from selected high-volume surgeons, and it makes predictions using a combination of theoretical optics, thin lens formulas, and ‘big data’ techniques. The Kane keratoconus formula has specific modifications for use in keratoconus patients. It uses a modified corneal power derived from anterior corneal radius of curvature that better represents the true anterior/posterior ratio in keratoconic eyes while also aiming to minimize the corneal power effect on the ELP calculation [16]. This may explain the accuracy of the Kane keratoconus formula over other formulas in keratoconic patients (Supplementary Materials Table S3).

Our analysis has several limitations. First, some studies did not measure the corneal posterior surface or higher-order aberrations, and this is beyond the scope of the study. Second, we did not include eyes with combined surgeries as refractive, cross-linking, or intracorneal rings segment procedures. Furthermore, it should be emphasized that only a limited number of studies have been published yet to compare some newer-generation formulas. For instance, the conclusion that the Kane formula may be preferable in estimating IOL power projection is based on only three studies. Finally, the optimal method to retrieve the K values themselves was beyond the scope of this study. 

## 5. Conclusions

In mild and moderate KC, cataract surgery results in satisfactory postoperative refraction. In advanced KC, both for non-toric and toric IOLs, postoperative refractive error remained high irrespective of the IOL formula used. Further research is warranted to optimize the IOL power prediction in advanced KC. As KC diagnosis has become more prevalent due to early detection, accuracy of IOL calculation formulas in such patients is now more relevant than ever. 

## Figures and Tables

**Table 2:** Mean error in non-toric and toric IOL power calculation in keratoconus stage I eyes based on Krumeich criteria.

Author	No. of Eyes	Year	Formula	Absolute Prediction Error SE (D)	Mean Prediction Error SE (D)	N/(%) of Eyes within 0.5 D	N/(%) of Eyes within 1 D
**Non-toric IOLs**							
Celikkol et al. [20]	1	1996	*SRK/T*	1.00	−1.00	0	1 (100)
Thebpatiphat et al. [6]	5	2007	*SRK II*	NA	+1.04	NA	NA
	4		*SRK*	NA	+1.42	NA	NA
	3		*SRK/T*	NA	+1.62	NA	NA
Watson et al. [21]	35	2013	*SRK/T*	NA	−1.1	NA	NA
**Toric IOLs**							
Navas et al. [22]	2	2009	*SRK II*	0.5	−0.5	2 (100)	2 (100)
Visser et al. [23]	3	2011	*SRK/T*	1.54	−1.54	1 (33)	2 (67)
Jaimes et al. [24]	15	2011	*SRK II*	0.61	−0.45	7 (47)	14 (93)
Nanavaty et al. [25]	8	2012	*Z calc*	0.25	+0.12	7 (88)	7 (88)
Parikakis et al. [26]	5	2013	*SRK/T*	0.95	−0.95	1 (20)	3 (60)
Alió e al. [27]	6	2014	*SRK/T*	0.25	+0.08	6 (100)	6 (100)
	7		*Hoffer Q*	0.93	−0.86	4 (57)	4 (57)
Hashemi et al. [28]	10	2015	*SRK/T*	0.81	NA	NA	NA
			*Holladay I*	0.89	NA	NA	NA
			*SRK II*	0.96	NA	NA	NA
			*Hoffer Q*	1.01	NA	NA	NA
Kamiya et al. [29]	14	2016	*SRK/T*	1.63	−1.63	10 (71)	14 (100)
**Unknown separation between non-toric and toric IOLs**							
Savini et al. [30]	21	2019	*SRK/T*	0.43 *	+0.44	13 (62)	17 (81)
			*Haigis*	0.61 *	+0.54	8 (38)	17 (81)
			*Barrett*	0.70 *	+0.63	9 (43)	16 (76)
			*Holladay I*	0.55 *	+0.75	9 (43)	17 (81)
			*Hoffer Q*	0.91 *	+0.90	5 (24)	13 (62)
Kane et al. [16]	84	2020	Kane (K*)	0.49	−0.18	51 (61)	76 (91)
			*Kane*	0.49	−0.18	51 (61)	76 (91)
			*Barrett*	0.54	−0.25	45 (54)	75 (89)
			*SRK/T*	0.56	−0.23	44 (52)	74 (88)
			*Holladay I*	0.56	−0.18	44 (52)	72 (86)
			*Hoffer*	0.57	−0.19	44 (52)	72 (86)
			*Haigis*	0.58	−0.26	43 (51)	72 (86)
			*Holladay II*	0.62	−0.38	39 (46)	69 (82)
			Holladay II (K*)	0.64	−0.36	32 (38)	68 (81)
Wang et al. [31]	46	2020	*Haigis*	0.58 *	+0.10	18 (39)	32 (70)
			*SRK/T*	0.62 *	+0.12	22 (48)	33 (72)
			*Holladay I*	0.65 *	+0.38	18 (39)	34 (74)
			*Barrett*	0.45 *	+0.39	24 (52)	35 (76)
			*Holladay II*	0.58 *	+0.56	18 (39)	33 (73)
			*Hoffer Q*	0.57 *	+0.65	19 (41)	31 (67)

* = median absolute difference between target and postoperative actual spherical equivalent (SE); K* = keratoconus adjustment; NA = not available.

**Table 3:** Mean error in non-toric and toric IOL power calculation in keratoconus stage II eyes based on Krumeich criteria.

Author	No. of Eyes	Year	Formula	Absolute Prediction Error SE (D)	Mean Prediction Error SE (D)	N/(%) of Eyes Within 0.5 D	N/(%) of Eyes Within 1 D
**Non-toric IOLs**							
Celikkol et al. [20]	1	1996	*SRK/T*	5.6	−5.6	0	0
Watson et al. [21]	40	2013	*SRK/T*	NA	−0.6	NA	NA
**Toric IOLs**							
Jaimes et al. [24]	4	2011	*SRK II*	1.84	−0.46	0	2 (50)
Nanavaty et al. [25]	4	2012	*Z calc*	0.4	+0.03	2 (50)	4 (100)
Alió e al. [27]	1	2014	*SRK/T*	1.25	−1.25	0	0
	1		*Hoffer Q*	2.50	−2.50	0	0
Hashemi et al. [28]	10	2015	*SRK/T*	0.69	NA	NA	NA
			*Holladay I*	0.78	NA	NA	NA
			*Hoffer Q*	0.80	NA	NA	NA
			*SRK II*	0.84	NA	NA	NA
Kamiya et al. [29]	5	2016	*SRK/T*	1.9	−1.5	3 (60)	4 (80)
**Unknown separation between non-toric and toric IOLs**							
Savini et al. [30]	13	2019	*SRK/T*	0.79 *	+0.54	4 (31)	7 (54)
			*Barrett*	1.42 *	+1.32	2 (15)	3 (23)
			*Holladay I*	1.23 *	+1.54	3 (24)	5 (38)
			*Hoffer Q*	1.55 *	+1.63	1 (8)	4 (31)
			*Haigis*	1.57 *	+1.66	2 (15)	2 (15)
Kane et al. [16]	37	2020	Kane (K*)	1.08	+0.53	16 (43)	21 (57)
			*SRK/T*	1.13	+0.51	11 (30)	18 (49)
			*Barrett*	1.21	+0.89	14 (38)	20 (54)
			*Kane*	1.23	+1.00	14 (38)	19 (51)
			*Holladay II*	1.27	+1.05	13 (35)	19 (51)
			*Holladay I*	1.31	+1.12	14 (38)	19 (51)
			*Haigis*	1.54	+1.34	6 (16)	16 (43)
			*Hoffer Q*	1.58	+1.47	6 (16)	18 (49)
			Holladay II (K*)	1.59	+1.41	6 (16)	15 (41)
Wang et al. [31]	22	2020	*SRK/T*	0.99 *	+0.36	4 (18)	11 (50)
			*Barrett*	0.45 *	+0.95	11 (50)	13 (59)
			*Haigis*	1.45 *	+1.12	5 (23)	8 (36)
			*Holladay I*	1.00 *	+1.21	9 (41)	11 (50)
			*Holladay II*	1.19 *	+1.49	5 (23)	10 (46)
			*Hoffer Q*	1.01 *	+1.70	4 (18)	11 (50)

* = Median absolute difference between target and postoperative actual spherical equivalent (SE); K* = keratoconus adjustment; NA = not available.

**Table 4:** Mean error in non-toric and toric IOL power calculation in keratoconus stage III eyes based on Krumeich criteria.

Author	No. of Eyes	Year	Formula	Absolute Prediction Error SE (D)	Mean Prediction Error SE (D)	N/(%) of Eyes Within 0.5 D	N/(%) of Eyes Within 1 D
**Non-toric IOLs**							
Watson et al. [21]	9	2013	*SRK/T*	NA	−0.6	NA	NA
**Toric IOLs**							
Hashemi et al. [28]	3	2015	*SRK/T*	0.39	NA	NA	NA
			*SRK II*	0.40	NA	NA	NA
			*Holladay I*	1.14	NA	NA	NA
			*Hoffer Q*	1.30	NA	NA	NA
**Unknown separation between non-toric and toric IOLs**							
Savini et al. [30]	7	2019	*Barrett*	2.65 *	+2.64	0	1 (14)
			*SRK/T*	3.99 *	+3.01	1 (14)	1 (14)
			*Haigis*	2.75 *	+3.26	0	1 (14)
			*Hoffer Q*	4.04 *	+3.46	1 (14)	1 (14)
			*Holladay I*	4.09 *	+3.77	0	0
Kane et al. [16]	25	2020	Kane (K*)	1.44	+0.02	6 (24)	12 (48)
			*SRK/T*	2.32	+1.86	3 (12)	5 (20)
			*Barrett*	2.45	+1.72	1 (4)	4 (16)
			*Kane*	2.64	+2.22	1 (4)	3 (12)
			*Haigis*	2.88	+2.43	1 (4)	4 (16)
			*Holladay II*	3.01	+2.62	2 (8)	3 (12)
			*Holladay I*	3.07	+2.43	1 (4)	3 (12)
			Holladay II (K*)	3.19	+2.88	2 (8)	4 (16)
			*Hoffer Q*	3.36	+3.02	0	3 (12)
Wang et al. [31]	5	2020	*Haigis*	1.18 *	+1.90	2 (40)	2 (40)
			*SRK/T*	1.74 *	+2.51	0	0
			*Holladay I*	1.96 *	+2.99	0	0
			*Hoffer Q*	4.22 *	+4.00	0	0
			*Holladay II*	4.52 *	+4.38	0	0
			*Barrett*	NA	NA	NA	NA

* = Median absolute difference between target and postoperative actual spherical equivalent (SE); K* = keratoconus adjustment; NA = not available.

**Table 5:** Weighted absolute prediction errors for top five IOL formulas in each KC stages.

	Formula	Absolute Prediction Error SE (D)	% of Eyes Within 0.5 D	% of Eyes Within 1 D
**Stage I**				
	Kane (K*)	0.49	61	91
	*Kane*	0.49	61	91
	*Barrett*	0.54	54	89
	*SRK/T*	0.73	54	83
	*SRK II*	0.73	53	94
**Stage II**				
	Kane (K*)	1.08	43	57
	*SRK II*	1.13	NA	NA
	*Holladay I*	1.20	38	51
	*SRK/T*	1.20	28	51
	*Barrett*	1.21	38	54
**Stage III**				
	Kane (K*)	1.44	24	48
	*SRK/T*	2.11	12	20
	*Barrett*	2.45	4	16
	*Kane*	2.64	4	12
	*Holladay I*	2.86	4	12

Absolute prediction error calculated from the studies reporting mean absolute difference between target and postoperative actual spherical equivalent (SE) having the data of at least 10 eyes. K* = keratoconus adjustment. NA = not applicable, data below 10 cases.

## Data Availability

The datasets analyzed during the current study are available from the corresponding author on reasonable request.

## Supplementary Materials

The following supporting information can be downloaded at: https://www.mdpi.com/article/10.3390/jcm11092456/s1, Figure S1: Flow diagram of inclusion process based on Preferred Reporting Items for Systematic Reviews and Meta-Analyses (PRISMA) guidelines [17]; Table S1: Krumeich criteria for *Classification of Keratoconus*; Table S2: Guidelines for target refraction in patients undergoing cataract surgery based on keratoconus (KC) grade by the Krumeich criteria; Table S3: IOL formula characteristics [16,41,42,43,44,45,46,47].

## Abbreviations

BCVA	best-corrected visual acuity
CXL	corneal cross-linking
D	diopter
ELP	effective lens position
IOL	intraocular lens
KC	keratoconus
