# Supplementary material for: Refractive Outcomes of Non-Toric and Toric Intraocular Lenses in Mild, Moderate and Advanced Keratoconus: A Systematic Review and Meta-Analysis
Source: J Clin Med. 2022 Apr 27;11(9):2456. doi: 10.3390/jcm11092456 (PMC9101494; doi:10.3390/jcm11092456)
Supplement: Supplementary file 1 [file jcm-11-02456-s001.zip › jcm-1619952-supplementary.pdf]

Refractive outcomes of non-toric and toric intraocular lenses in mild, moderate, and advanced keratoconus: a systematic review and meta-analysis

Tal Yahalomi, MD[1]; Asaf Achiron, MD[2]; Idan Hecht, MD[2,3]; Roee Arnon, MD[1]; Elyia Levinger[2,4], MD, Joseph Pikkel, MD[1]; Raimo Tuuminen, MD, PhD[5,6]

[1]Department of Ophthalmology, Samson Assuta Ashdod Hospital, Faculty of Health Sciences, Ben-Gurion University of the Negev, Israel
[2]Sackler Faculty of Medicine, Tel Aviv University, Tel Aviv, Israel
[3]Department of Ophthalmology, Shamir Medical, Zerifin, Israel
[4]Enaim Refractive Surgery Center, Jerusalem, Israel
[5]Department of Ophthalmology, Kymenlaakso Central Hospital, Kotka, Finland
[6]Helsinki Retina Research Group, Faculty of Medicine, University of Helsinki, Helsinki, Finland

Short title: Refractive outcomes in keratoconus

Supplement Figure S1.
Flow diagram of inclusion process based on Preferred Reporting Items for Systematic Reviews and Meta-Analyses (PRISMA) guidelines[17].

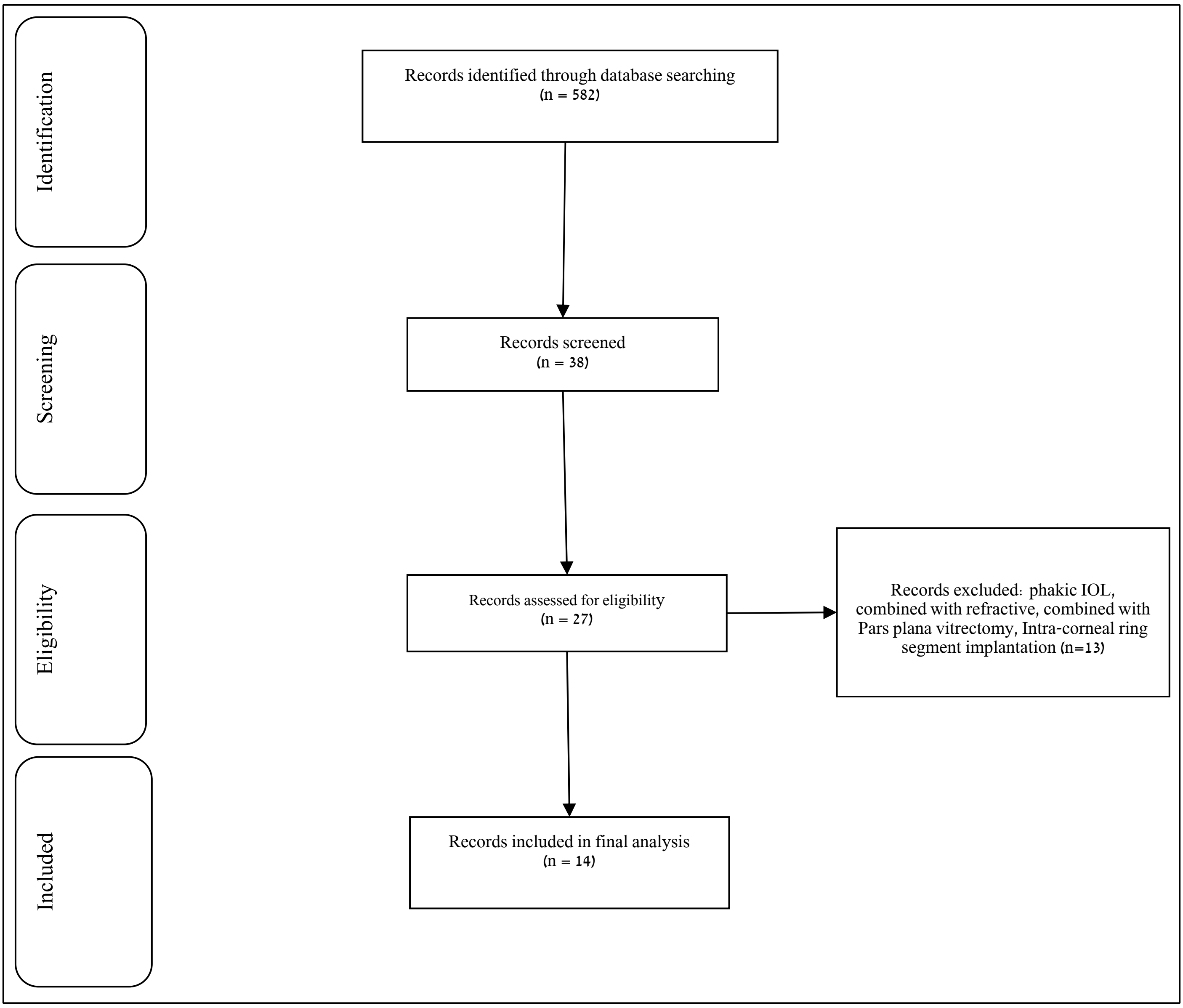

Supplement Table S1. Krumeich criteria for *Classification of Keratoconus.*

Stage	Keratometry (mean K)
I	< 48 D
II	48 - 53 D
III	> 53 D
IV	unmeasurable refraction or with a central scar

Supplement Table S2. Guidelines for target refraction in patients undergoing cataract surgery based on keratoconus (KC) grade by the Krumeich criteria.

**KC grade**	**Watson et al. [25]**	**Kane et al. [11]**
Stage I	-1.0 D	No adjustment
Stage II	-1.5 D	Between -0.75 and -1.5 D
Stage III	Actual K values may result in significant hyperopic surprise; hence the use of standard K values should be explored. This grade has no refractive aim recommendation.	Between -2.0 and -3.0 D

Supplement Table S3: IOL formula characteristics.

IOL power formula	Parameters used for ELP prediction	Recommended eye type	Description
**Barrett**	AL, corneal power, ACD (optional), lens thickness (optional), white-to-white corneal diameter (optional).	Recommended for short – long eyes.	The important difference between formulas is that the location of the principle plane of refraction of the IOL is retained as a relevant variable in the formula(1).
**Haigis**	AL and ACD.	Haigis (a_0 optimized only): normal eyes. Haigis (a_0, a_1, a_2 optimized): short – long eyes	Based on a three-variable (a0, a1 and a2) function.The measured ACD is linked to the a1 constant, while the measured AL is tied to the a2 constant. Using double-regression analysis, all three constants may be optimized for a wide variety of ALs and ACDs(2).
**Hoffer Q**	AL and corneal power	Used for eyes measuring < 22 mm	Based on a personalized ACD, AL, and corneal curvature. Any series of an IOL style can be used to create the personalized ACD. It contains a factor that increases ACD as AL increases, a factor that increases ACD when corneal curvature increases, a factor that moderates the change in ACD for exceptionally long and short eyes, and a constant added to the ACD(3).
**Holladay I**	AL and corneal power	Used for eyes measuring between 24.6 mm – 26.0 mm	Calculates a tailored surgeon factor using the postoperative refraction value, the dioptric power of the implanted IOL, and the preoperative corneal and AL measurement. The distance between the post-operative anterior iris plane and the effective optical plane of the IOL is thus termed as the surgeon factor (4).
**Holladay II**	AL, corneal power, ACD, lens thickness (optional), age (optional), white to-white corneal diameter (optional), pre-operative refraction data (optional).	Recommended for short – long* eyes.W-K adjustment has been shown to extend the usage from "short – normal eyes" to "short – long eyes" for meniscus IOLs designs in the low plus to minus power range(2).	Similar to the Holladay I method, but it predicts the surgeon factor using seven parameters: AL, corneal power, ACD, lens thickness, age, white-to-white corneal diameter, and pre-operative refraction.
**SRK, SRKII**			The SRK I and SRK II formulas are obsolete and should no longer be used(5).
**SRK/T**	AL and corneal power	Used for eyes > 26 mm. A combination of	Under the SRK umbrella of empirical formulae, the SRK/T

		Hoffer Q, Holladay 1, and SRK/T should be used for eyes 22.0–24.5 mm	formula is using a known A-constants and optimization approaches. The SRK/T model's empirical optimization approaches include postoperative ACD prediction, a retina thickness correction factor, and corneal refractive index(6).
**Kane**	AL, keratometry, ACD, CCT and gender	Long eyes (26.0 mm) and short eyes (22.0 mm).	Kane formula in refractive prediction was comparable in IOL power calculation, marking its superiority over many conventional IOL formulas, such as HofferQ, Haigis, Holladay1, and Holladay2 (7).
**Kane (K*)**	AL, keratometry, ACD, CCT and gender	Keratoconus	Available at www.iolformula.com. It uses a modified corneal power derived from anterior corneal radius of curvature that better represents the true anterior/posterior ratio in keratoconic eyes while also aiming to minimize the corneal power effect on the ELP calculation(8).
**Z calculator**	AL, keratometry and ACD	Toric IOL	A calculation of the ZEISS toric IOLs ZEISS premium refractive toric IOLs , Access Z CALC at www.iolmaster-online.zeiss.com

AL; axial length, ACD; anterior chamber depth, CCT; central corneal thickness. K*; keratoconus adjustment.

1. Barrett GD. An improved universal theoretical formula for intraocular lens power prediction. J Cataract Refract Surg [Internet]. 1993 [cited 2022 Apr 13];19(6):713–20. Available from: https://pubmed.ncbi.nlm.nih.gov/8271166/
2. IOL Power Calculations Which Formula? [Internet]. [cited 2022 Apr 13]. Available from: https://doctor-hill.com/
3. Hoffer KJ. The Hoffer Q formula: A comparison of theoretic and regression formulas. J Cataract Refract Surg [Internet]. 1993 [cited 2022 Apr 13];19(6):700–12. Available from: https://journals.lww.com/jcrs/Fulltext/1993/11000/The_Hoffer_Q_formula__A_comparison_of_theoretic.7.aspx
4. Holladay JT, Musgrove KH, Prager TC, Lewis JW, Chandler TY, Ruiz RS. A three-part system for refining intraocular lens power calculations. J Cataract Refract Surg. 1988 Jan 1;14(1):17–24.
5. Németh J, Fekete O, Pesztenlehrer N. Optical and ultrasound measurement of axial length and anterior chamber depth for intraocular lens power calculation. J Cataract Refract Surg [Internet]. 2003 Jan 1 [cited 2022 Apr 13];29(1):85–8. Available from: https://pubmed.ncbi.nlm.nih.gov/12551672/
6. Retzlaff JA, Sanders DR, Kraff MC. Development of the SRK/T intraocular lens implant power calculation formula. J Cataract Refract Surg [Internet]. 1990 [cited 2022 Apr 14];16(3):333–40. Available from: https://pubmed.ncbi.nlm.nih.gov/2355321/
7. Ryu S, Jun I, Kim TI, Kim EK, Seo KY. Accuracy of the Kane Formula for Intraocular Lens Power Calculation in Comparison with Existing Formulas: A Retrospective Review. Yonsei Med J [Internet]. 2021 Dec 1 [cited 2022 Apr 13];62(12):1117. Available from: /pmc/articles/PMC8612861/

8. Kane JX, Connell B, Yip H, McAlister JC, Beckingsale P, Snibson GR, et al. Accuracy of Intraocular Lens Power Formulas Modified for Patients with Keratoconus. In: Ophthalmology [Internet]. Elsevier Inc.; 2020 [cited 2021 Jan 28]. p. 1037–42. Available from: https://pubmed.ncbi.nlm.nih.gov/32279887/
